# Home × office or home and office: importance of breaks at work

**DOI:** 10.47626/1679-4435-2023-857

**Published:** 2023-04-18

**Authors:** Carla Aparecida Spagnol

**Affiliations:** 1 Nursing School, Universidade Federal de Minas Gerais, Belo Horizonte, MG, Brazil

**Keywords:** teleworking, COVID-19, occupational health, personnel management, health promotion, teletrabalho, COVID-19, saúde do trabalhador, administração de recursos humanos, promoção da saúde

## Abstract

The COVID-19 pandemic can be seen as a rite of passage where individuals are being called upon to rethink the dictatorship of the way-of-being through work. With the intensification of the *work from home* modality, many essential aspects of life became secondary. This way, it is important to think about work breaks, not only from the viewpoint of labor laws, but also for creating moments of reflection for (re)thinking various aspects of work, whether remote or in person. The objective of this study was to promote a reflection on the importance of taking breaks during remote work (working from home) or in-person work, considering the promotion of occupational health and well-being. Breaks during the workday are beneficial to physical and mental health, as they help restore concentration and energy, relieve stress, improve muscle tension, among other factors. Strategies for promoting work breaks cannot be prescribed as recipes but should be considered as possibilities to exercise these moments of disconnection from work on a daily basis. Moreover, the worker can also contribute to improve the quality of working life by adopting simple attitudes such as maintaining adequate hydration and using practices such as foot soaks, meditation, yoga, self-massage, foot reflexology, and mindfulness in the work environment. Therefore, in order for strategies for the promotion of health and occupational well-being to be successful, we need a change in the behavior of managers and workers in order to better reconcile our way-of-being through work and our way-of-being through care.

## Abstract (pt)

A pandemia da COVID-19 pode ser vista como um rito de passagem em que os indivíduos estão sendo convocados a repensar a ditadura do modo-de-ser trabalho. Com a intensificação da modalidade do *home office*, vários aspectos essenciais da vida ficaram em segundo plano. Assim, é importante pensar acerca da pausa laboral, não só a partir da legislação trabalhista, mas também criando momentos de reflexão para (re) pensar vários aspectos do trabalho, seja remoto ou presencial. O objetivo deste artigo foi promover uma reflexão acerca da importância da pausa no trabalho remoto (*home office*) ou presencial, tendo em vista a promoção da saúde e o bem-estar ocupacional. As pausas durante a jornada de trabalho trazem benefícios para a saúde física e mental, pois ajudam a restaurar a concentração, revigorar as energias, aliviar o estresse, melhorar as tensões musculares, entre outros. As estratégias para promover a pausa no trabalho não podem ser prescritas como receitas, mas esses momentos de desconexão com o trabalho devem ser considerados como possibilidades de se exercitar cotidianamente. Além disso, o trabalhador também pode contribuir para melhorar a qualidade de vida no trabalho ao adotar atitudes simples como manter uma hidratação adequada e utilizar práticas como escalda-pés, meditação, ioga, automassagem, reflexologia podal e *mindfulness* no ambiente laboral. Portanto, para que as estratégias de promoção à saúde e de bem-estar ocupacional obtenham sucesso, é necessário que haja uma mudança de comportamento de gestores e trabalhadores a fim de conciliar melhor nosso modo-de-ser trabalho e nosso modo-de-ser cuidado.

## INTRODUCTION

### THE PANDEMIC AS A RITE OF PASSAGE

Since the beginning of 2020, the effects of the COVID-19 pandemic caused by the SARS-CoV-2 are affecting society in its political, economic, social, and cultural aspects. Many changes have taken place in this context, such as distancing between people, public health actions, emergency economic measures, the devastating number of deaths, increase in unemployment, and remote work and education.^1,2^

Considering these changes, in this article we chose not to bring numbers or statistics, but instead reflect on the importance of breaks at work, whether remote (working from home) or in-person, especially in the context of the pandemic. For this, we sought inspiration in the book “Essential care: an ethics of human nature,” written by Leonardo Boff in 1999,^3^ and other important literature in this theme. What Boff wrote then is still relevant considering what we are going through today.

In 1999, the author already said: “We are not experiencing the end of the world, we are in fact experiencing the end of a type of world. We are facing a crisis affecting human civilization (...)”. Therefore, “We are in need of a new paradigm for living together (...). Only if these changes occur will it make sense for us to start thinking about alternatives that may present us with new hope.”^3^ This citation makes us think that, even if metaphorically, a “virus’ genetic mutation” had to happen to lead to a so-called “mutation” in society and the search for a “new hope” to build a new type of world.

The pandemic can thus be seen as a rite of passage rather than as an apocalypse in the religious sense of the word, instead as a moment of rupture marked by important changes in people’s lives.

This regard of the pandemic as a rite of passage allows us to understand that individuals are being called upon to rethink life, especially when it comes to the dictatorship of the way-of-being through work, as in recent times our lives have been revolving around work 24 hours a day, depriving us of many other essential aspects such as family, leisure, friendships, our own health - things that are many times left aside due to work.

This was intensified with the COVID-19 pandemic, as remote work is occupying and sharing space in the home environment with other activities, which previously had clearer boundaries. This intensification can lead to a decrease in spaces and moments dedicated to idleness, family interaction, rest, and physical and mental rehabilitation.^4^

Working from home stood out among remote work modalities, especially in the current context of the pandemic. Working from home started in the 1970s and constitutes a flexible work modality performed at the worker’s home; he or she mainly uses the Internet for developing the activities of his or her work routine.^5,6^

Some authors point out that working from home has provided a share of the global population with opportunities of keeping their jobs, with the advantage that workers are able to protect themselves from contamination with the new coronavirus by reducing social contact and consequently mass contagion, avoiding the collapse of health systems.^4^

On the other hand, some disadvantages are more recurrent, such as the conflict between personal life and work issues, greater professional and social isolation, difficulty controlling the workload, psychological impacts, low motivation, and increased home utility costs - for example, electricity, Internet, telephone, and water bills, among other expenses.^5^

Along with the aforementioned issues, another obstacle is managing time and space boundaries when working at home. Therefore, one of the most common consequences of working from home is a longer workday than the one usually required when working in person. With this, the worker has difficulties maintaining a routine and many times ends up losing control of the time and space reality in this new “work environment” - his or her own home.^7^

In this perspective, the workload of people working from home should be noted, as it is usually 10 to 20% larger than that of in-person employees. In addition, for some authors, the flexibility of the work routine can camouflage the intensification of work.^5,8^

In remote work, “despite the competition between household chores or family interaction and the time spent working, the individual remains online and responding, meeting performance goals and deadlines and avoiding any impression of idleness on his or her hours. Therefore, by removing the employer’s oversight of a minimum workday to be performed by the subordinates (attendance control), teleworking may amplify the employer’s power, as it is no longer restricted to registering the time an individual spent in a certain environment but instead turns every space this person may occupy into a work environment.”^4^

According to Boff, the great challenge is to combine work and care and understand that these two ways-of-being in the world are not opposites, but should comprise each other. For the author, “The recovery of the way-of-being through care is not achieved to the detriment of work; it is rather done through a different way of understanding and performing work. In order to do this the human being needs to turn itself to itself and discover its way-of-being through care.”^3^

This way, it is important to think about work breaks, not only from the viewpoint of labor laws, but also considering the creation of moments of reflection for (re)thinking various aspects of work, whether remote or in person. At this point of our work, rather than answers, many questions have emerged:

1. How to organize new forms of work? Shouldn’t we better use the available technologies and invest in others?
2. Work must be performed and quality products must be delivered. However, shouldn’t we have better working conditions to reduce occupational risks?
3. How to enable leadership development? It can be said that many people in leadership roles still centralize decisions and use subtle or even invisible forms of violence, harassment, and oppression with their teams.
4. How to educate autonomous, nonsubmissive professionals, with skills and competencies for achieving collective decision-making?
5. Is it possible to work more creatively and with freedom to propose individual and collective ideas and projects?

Creating breaks for collectively reflecting on working conditions and interpersonal relationships at work may help us better understand work (seen here as the “office”) and its modalities. It may also make us think how to seek strategies for promoting workers’ health and well-being.

Considering these aspects, the objective of this article was to promote a reflection on the importance of breaks during remote work (working from home) or in-person work, considering the promotion of occupational health and well-being.

### IMPORTANCE OF BREAKS AT WORK

Breaks for reflecting upon new ways of working and new conducts are as needed as those stipulated by labor laws. It is thus important to shed light on the need to care for workers and for them to practice self-care - as “home” is our house but also our body, which requires care.

In this sense, people should be called upon rescuing care as one of the dimensions of the human being, in its way-of-being through care. This is due to the existence of something in human beings that is not found in machines: the feeling, the ability to be moved, involved, affected, and to affect.^3^

Therefore, breaks throughout the workday are required both for remote and in-person work. From time to time the body needs to stop or else workers, which are human beings and not machines, fall ill!

The types of breaks stipulated by labor laws must be recognized. The daily rest break lasts at least 11 hours between workdays; workday breaks last up to 15 minutes for those who work 6 hours and at least 1 hour for those who work 8 hours a day.^9,10^

Workers frequently forget to care for themselves during the workday. For example, health professionals working in-person at sectors dedicated to caring for patients with COVID-19. These professionals have great difficulty taking breaks during their workday, as they need to remove and put on personal protective equipment, which is not easy and takes time. With this, they frequently end up forgetting to take care of themselves. In this case, the question is: if health professionals do not care for themselves, how will they be well enough to care for other people?

This question is directed not only to health professionals but also to overall workers, especially those who are currently working at home; these are more prone to having their domestic and personal lives invaded by work issues.

This way, technological advances have significantly contributed to speeding up work activities, but at the same time have made us obsessed with information by checking e-mails or text messages outside of the work environment, even without realizing it, which prevents us from disconnecting when off work.^11^

In the context of the pandemic, we observed that when faced with the power of companies and “permeated with a pretended ‘meritocracy’ at the expense of personal sacrifice, a considerable number of employees working remotely is deprived of the right to disconnect from their jobs, working long hours and leading to physical and mental exhaustion, being at risk of chronic stress, burnout, or depression.”^12^

This is why planning working hours and acquiring a certain discipline is important. This is true for in-person work, but especially for remote work (working from home), where workers currently divide their time with other household chores and their children’s homework, all in the same environment. This exercise of discipline and time management is extremely necessary for not losing sight of the way-of-being through care.

Therefore, it is vital to think about health promotion and self-care and, in this regard, other issues appeared in our reflection:

1. What do we do in life with our health?
2. Which leaders are taking care of their teams during remote work in this pandemic?
3. How do workers organize for taking breaks during their workday?

It is thus of utmost importance that workers be able to practice self-care, but organizations also need to ensure their rest in order to protect their physical and psychological health, avoiding work overload and respecting the workers’ family environment and leisure time.^12^

Breaks during the workday need to be understood as beneficial to physical and mental health, as they help restore concentration and energy, relieve stress, improve muscle tension, among other factors.

The literature indicates some individual or collective strategies for caring for the body and enabling work breaks. Some organizations such as Google, Target, and the United States Navy have provided alternative treatments for managing stress, introducing meditation sessions during the workday.^11^

Many other organizations also use physical exercises, workplace physical activity, and educational games, in addition to many complementary and integrative health (CIH) practices that were made official by the Ministry of Health, such as music therapy, yoga, foot soaks, and mindfulness. Studies indicate that these practices significantly contribute to raise self-esteem, increase productivity, and improve workers’ disposition and satisfaction. In addition, they may increase stress tolerance, decrease absenteeism, and improve the teams’ interpersonal relationships, which contributes to the quality of the performed work.^13-16^

These strategies for promoting work breaks cannot be prescribed as recipes but should be considered as possibilities to exercise these moments of disconnection from work on a daily basis. Moreover, the worker contributes to improving the quality of working life by adopting simple, but essential attitudes, such as maintaining adequate hydration and before starting work, during the workday, or even after work, using some of these CIH practices: foot soaks, meditation, yoga, self-massage, foot reflexology, or mindfulness.

According to Boff, “caring for the body means to search for the creative assimilation of all that may occur to us in life, commitments and work, meaningful encounters and existential crises, successes and failures, health and suffering. Only then can we turn into ever more mature, autonomous, wise, and fully free people.”^3^

This is why for strategies related to the promotion of health and occupational well-being to be successful, we need a change in the behavior of managers and workers in order to better reconcile our way-of-being through work and our way-of-being through care. Creating moments of reflection is also necessary, inviting managers and their teams to think about the concepts of atmosphere, interpersonal relationships, and (re)organization of the work process, in order to intervene with the institution’s micro and macro policies and aiming for improvements in working conditions and occupational health.

## FINAL CONSIDERATIONS

With these reflections, one could say that, both for remote work (working from home) and in-person work, we need to create permanent spaces of analysis of the professional activity that allow us to understand the three dialectical moments of the work institution.

The instituted moment is understood as in-person work and the home office, that already existed in some areas before the pandemic, in which workers already had an established work routine.

The institutor moment is considered the form of remote work that, during the pandemic, was suddenly instituted in various areas and did not allow workers to plan their work activities, which invaded household chores; therefore, workers and their family members are living with “home vs office.”

The moment of institutionalization comprises what may come to be the “home” (body) and the “office” (work), that is, new forms of work that may include productivity, self-care, and caring for workers and professional relations.

In conclusion, one may think that the home office and “home vs office” could be replaced by “home” and “office.” Is this reflection possible?
